# The Race of Life

**Published:** 1885-08

**Authors:** 


### THE RACE OF LIFE.

WE take the following from the pages of as handsome and sprightly a new Journal as is gotten up in this Country, the second number of volume one of *The Peoples' Health Journal*, published at Chicago. It is printed on the best quality of extra heavy S. & S. C. paper, royal octavo in size, double column, in clean new long primer type; and is edited by L. D. Rogers, A. M., M. D., and S. Ida Wright Rogers, M. D. We welcome it to our exchange list, and will have something more to say of it, bye and bye.

"A Sporting journal thus describes the race of life: "If one could see a million babies start out on a journey (all scratch the mark of cause) and could follow them through life, this is about what he would see: Nearly 150,000 of them drop out of the ranks by the end of the first year; while twelve months later the numbers would be further thinned by the deduction of 53,000 more; 28,000 would follow at the end of the thirteenth year. They would throw up the sponge by twos and threes until the forty-fifth year, when it would be found that in the intervening period something like 500,000 had left the track. Sixty years would see 370,000 gray-headed men still cheerfully pegging away. At the end of eighty years the competitors in this great go-as-you-please, would number 97,000, but they would be getting more shaky and 'doty' each lap. At the end of ninety-five seasons, 223 would only be left in the final 'ties,' while the winner would be led to his resting room, a solitary wreck, at the age of one hundred and eight."

"There is something grimly humorous in this quaint array of figures, but they are founded upon statistics carefully compiled. One cannot help but wonder what would be the betting, at the start, about any one of those million babies coming in alone at the one-hundredth lap of the great and myterious track upon which the race of life is run."

